# Small RNA populations revealed by blocking rRNA fragments in *Drosophila melanogaster* reproductive tissues

**DOI:** 10.1371/journal.pone.0191966

**Published:** 2018-02-23

**Authors:** Emily K. Fowler, Irina Mohorianu, Damian T. Smith, Tamas Dalmay, Tracey Chapman

**Affiliations:** 1 School of Biological Sciences, University of East Anglia, Norwich Research Park, United Kingdom; 2 School of Computing Sciences, University of East Anglia, Norwich Research Park, United Kingdom; Niels Bohr Institute, DENMARK

## Abstract

RNA interference (RNAi) is a complex and highly conserved regulatory mechanism mediated via small RNAs (sRNAs). Recent technical advances in high throughput sequencing have enabled an increasingly detailed analysis of sRNA abundances and profiles in specific body parts and tissues. This enables investigations of the localized roles of microRNAs (miRNAs) and small interfering RNAs (siRNAs). However, variation in the proportions of non-coding RNAs in the samples being compared can hinder these analyses. Specific tissues may vary significantly in the proportions of fragments of longer non-coding RNAs (such as ribosomal RNA or transfer RNA) present, potentially reflecting tissue-specific differences in biological functions. For example, in *Drosophila*, some tissues contain a highly abundant 30nt rRNA fragment (the 2S rRNA) as well as abundant 5’ and 3’ terminal rRNA fragments. These can pose difficulties for the construction of sRNA libraries as they can swamp the sequencing space and obscure sRNA abundances. Here we addressed this problem and present a modified “rRNA blocking” protocol for the construction of high-definition (HD) adapter sRNA libraries, in *D*. *melanogaster* reproductive tissues. The results showed that 2S rRNAs targeted by blocking oligos were reduced from >80% to < 0.01% total reads. In addition, the use of multiple rRNA blocking oligos to bind the most abundant rRNA fragments allowed us to reveal the underlying sRNA populations at increased resolution. Side-by-side comparisons of sequencing libraries of blocked and non-blocked samples revealed that rRNA blocking did not change the miRNA populations present, but instead enhanced their abundances. We suggest that this rRNA blocking procedure offers the potential to improve the in-depth analysis of differentially expressed sRNAs within and across different tissues.

## Introduction

RNA interference (RNAi), is a complex and highly conserved gene regulatory mechanism [1,2] mediated via small RNAs (sRNAs). Based on their biogenesis and mode of action, sRNAs are classified into microRNAs (miRNAs) and small interfering RNAs (siRNAs). miRNAs are derived from a single-stranded RNA, have a hairpin-like secondary structure and regulate gene expression at the post-transcriptional level. siRNAs are excised from a double-stranded RNA and can act at transcription and post-transcription [3]. sRNAs play an important role in plants [4], animals [5] and fungi [6] in gene regulation or defence against pathogens.

The state of the art for the identification and characterization of sRNA populations is sRNA sequencing (sRNA-seq). Recent technical advances in high throughput sequencing [7,8] resulting in increased sequencing depth and resolution, have enabled the analysis of more complex datasets and a focus on describing the sRNA populations in specific tissues of interest. For example, in *D*. *melanogaster*, it is now known that the sRNA population comprises miRNAs (22mers, with 21nt and 23nt sequence variants), siRNAs (21mers, with 20nt and 22nt variants) and piRNAs (~29-30nt long) [9]. Different cell types may also contain variable ratios of different sRNAs [9].

sRNA-seq libraries can be prepared from either total RNA, or RNA which has been enriched for short fragments. Both methods require further size selection of the ~20–30 nt fraction via either manual gel extraction, or automated size selection (using the BluePippin system). One of the biggest obstacles in generating an informative sRNA-seq output is the variable proportion of reads derived from ribosomal RNAs (rRNAs). In plants and animals, the mature 18S, 5.8S and 25/28S rRNAs are processed from a long, polycistronic transcript [10]. The shortest of these rRNAs, the 5.8S, is processed in *Drosophila*, and at least some other Diptera [11], to produce a 30 nt 2S rRNA [12]. rRNAs can be present in sRNA samples either as random degradation products from longer rRNAs, or intact short rRNAs, e.g. the 2S rRNA [12,13]. Variation in the ratio of sRNAs to rRNAs may reflect biological differences between different tissues [14].

The size and high abundance of 2S rRNA can interfere markedly with the construction of sRNA libraries in *D*. *melanogaster*, since the size selection or enrichment for small fragments also captures the 2S rRNA fragments. For example, previous studies [15] reported >95% of sRNA-seq reads corresponding to 2S rRNA. This level of rRNA contamination can severely compromise the quantification of sRNA populations as it swamps the sequencing space. For example, at this level of rRNA contamination, with an average sequencing depth of 10 million reads per sample, <500,000 reads would correspond to miRNAs, leading to additional problems in achieving the minimum sequencing depth required for rigorous quantitative analysis. To gain sufficient depth for the analysis of sRNA abundances, one solution is to increase the overall sequencing depth by multiplexing fewer samples per sequencing lane. However, this significantly increases the sequencing cost per sample and is inefficient because most of the sequencing space is taken up by the contaminating rRNA fragments.

Since sRNAs are not poly-adenylated, rRNAs cannot be excluded through polyA extraction methods as for mRNA-seq libraries [16]. Alternative methods of rRNA removal, such as Ribozero and RNaseH treatments are expensive or involve additional steps in the library protocol, each of which can potentially alter the sRNA populations. An alternative approach is to apply a blocking step to remove the most abundant rRNA fragments. This has been used successfully to deplete 2S rRNA to <0.1% of reads in RNA extracted from *Drosophila virilis* ovarian tissue [17]. Here, we apply and further develop this approach, by: (i) modifying blocking oligos to prevent adapter ligation, (ii) simplifying protocols by adding blocking oligos directly to the extracted RNA, and (iii) applying a novel ‘oligo cocktail’ to selectively target multiple abundant rRNA fragments.

We first conducted sRNA sequencing on *D*. *melanogaster* head+thorax (HT) and abdomen (AB) samples in females, using libraries constructed with high definition adapters [7]. This analysis highlighted a problem with an overabundance of rRNA fragments, particularly in abdomen tissues. We successfully addressed this, using abdomen samples derived from males, by applying a modified, single oligo rRNA blocking approach [17] to remove the most abundant 30mer 2S rRNA. However, in some tissues, such as the male accessory glands and testes (AGT), the population of rRNA fragments appeared more complex. In this situation, blocking with a single oligo was not effective. Using accessory gland (AG) tissues, we demonstrated that the use of a blocking oligo ‘cocktail’ to simultaneously target multiple, abundant rRNA fragments, increased the overall abundance of sRNA reads. In addition, there was good agreement between the sRNA populations before and after blocking, suggesting that this procedure did not introduce any detectable bias. We conclude that the multiple oligo blocking method can provide a rigorous description of the complex populations of sRNAs in specific tissues, facilitating their comparison.

## Results

### Sequencing quality check of sRNAs in the sequenced samples

The initial quality check of the sequencing output for all the sRNA-seq samples used in this study indicated good technical scores (S1 Table). Fewer than 0.1% of the reads contained unassigned nucleotides (nt) and for > 96% of reads the 3’ adapters were trimmed based on a perfect sequence similarity with the first 7nt of the adapter. After the additional trimming of the HD signatures (4 nt at the 5’ and 3’ end of each insert) > 85% reads represented valid inserts and were retained for the subsequent analyses. These results suggest that the sequencing output was reliable. The overall sample complexities [18], defined as the ratio of redundant to non-redundant reads was variable (ranging from 0.005 to 0.125), yet low, indicating the presence of a relatively small number of highly abundant reads. Consistent with this, checks using the available annotations (*D*. *melanogaster* miRNAs from miRbase [19] and rRNAs [20]) indicated an over-representation of rRNA fragments.

### The proportion of 2S rRNA reads was highly variable across body parts and tissues

To describe the sRNA populations in different *D*. *melanogaster* tissues, RNA enriched for short fragments (<200 nt) was extracted from 50 pooled female head/thorax (HT_f_) or abdomen (AB_f_) body parts (see Materials and Methods). To reduce the ligation bias of T4 RNA ligase, ‘High Definition’ (HD) adapters [7], consisting of 4 degenerate nucleotides at the ligating ends of the adapters, were used to construct cDNA libraries from the extracted material, as described in [8].

**Fig 1:**
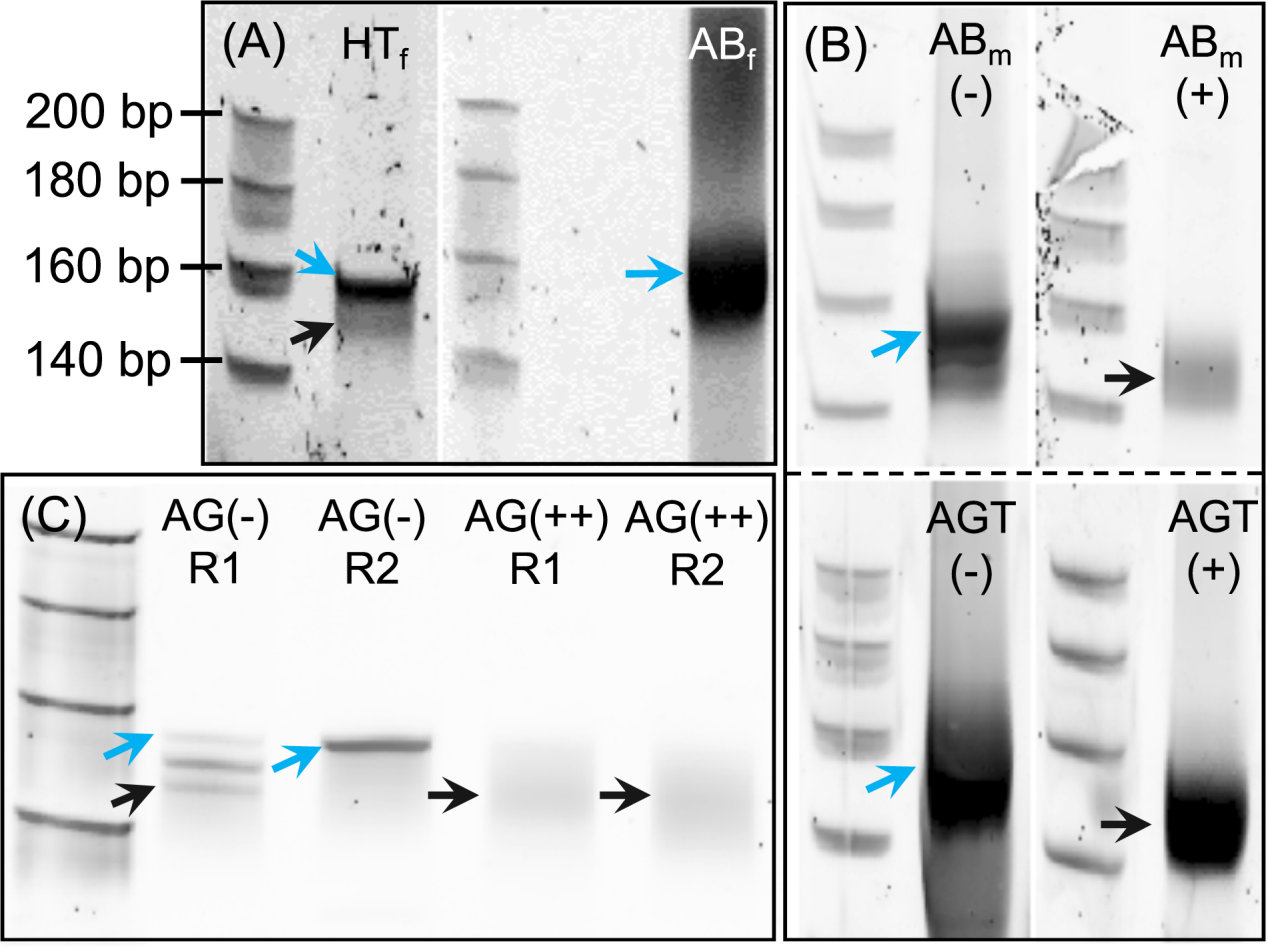
Size separation of major RNA bands in cloned cDNA libraries. Shown are 8% polyacrylamide gels (size ladder is the same across all gel images). Bands containing cDNA inserts of 21–22 nt are indicated with black arrows, and blue arrows indicate 30 nt inserts. (A) Standard libraries from female head/thorax (HT_f_) and abdominal (AB_f_) tissue. (B) Libraries of male abdominal tissue (AB_m_), and accessory glands/testes (AGT), made using standard protocol without blocking oligos (-), or a 2S rRNA blocking oligo (+). (C) Libraries of male accessory glands (AG), made using standard protocol without blocking oligos (-), or multiple rRNA blocking oligos (++). R1 and R2 indicate biological replicates. For AG- R1, a third band was visible, corresponding to an insert size of 26 nt (as also observed in the sequencing data).

Following adapter ligation and RT-PCR of the libraries, the amplified products were separated on 8% polyacrylamide gels (Fig 1A). Cloned libraries of HT_f_ tissue migrated as two distinct bands, corresponding to insert sizes of 21–22 nt, and 30 nt. Contrastingly, only the 30 nt band was visible for the AB_f_ libraries. Inserts of 30 nt in *D*. *melanogaster* RNA libraries typically include 80–90% mature 2S rRNA, which is processed from pre-ribosomal rRNA as a 30 nt long fragment [12]. We excised the region containing 21–22 nt fragments from each gel and purified, quantified and sequenced it.

Initial quality checks of the sequencing data (S1 Table) revealed a low overall sample complexity (defined as the ratio of non-redundant to redundant reads) for both tissues, indicating the presence of a relatively small number of highly abundant reads. Annotation analysis revealed that >92% of reads mapped to *D*. *melanogaster* genome (Table 1, Table 2). In the HT_f_ sample, 50.7% of reads were annotated as miRNAs, and 40.1% aligned to rRNA Contrastingly, for the AB_f_ sample, miRNAs made up only 3.6% of redundant reads, while the vast majority (86.7%) matched to rRNAs. In each tissue, the majority of rRNA reads (39.7% (HT_f_) and 84.3% (AB_f_)) mapped to the 30nt 2S rRNA, reflecting the dominant 30nt band observed following electrophoresis (Fig 1A and 1B, Table 2).

**Table 1:** Annotation analysis of genome, miRNA and rRNA mapping reads, for the blocked and non-blocked sRNA-seq libraries. Shown for each sample are the number of redundant (R) and unique non-redundant (NR, unique) reads, the overall complexities (ratio of NR:R) and the proportion (Prop) of R and NR reads incident with each annotation. HT = head+thorax; AB = abdomen (subscripts f and m for female and male, respectively); AGT = male accessory glands + testes; AG = male accessory glands. For the AB_m_ samples, for the non-blocked treatment, 89.5% of reads matched to rRNAs and only 7.6% to miRNAs. When the single oligo blocking was used, 47% of reads matched to miRNAs. For the AGT samples, 99% of reads matched to rRNAs in the non-blocked samples and 86.4% to rRNAs when the single oligo blocking was used. The proportion of miRNA annotated reads was 2.6%. For the AG tissue, the blocking increased the miRNA population from < 1% to 5.7% and 7.6%.

			Genome matching reads					rRNA matching reads					miRNA matching reads				
Sex	Treatment	Sample	R	NR	C	Prop R	Prop NR	R	NR	C	Prop R	Prop NR	R	NR	C	Prop R	Prop NR
Female	not-blocked	HTf non-blocked	11,147,369	125,717	0.011	0.921	0.452	4,860,253	44,448	0.009	0.401	0.160	6,134,942	2,972	0.000	0.507	0.011
Female	not-blocked	ABf non-blocked	19,357,042	152,845	0.008	0.928	0.520	18,090,977	44,896	0.002	0.867	0.153	744,322	1,789	0.002	0.036	0.006
Male	single oligo	ABm blocked	2,421,120	262,527	0.108	0.316	0.273	681,409	76,987	0.113	0.281	0.293	1,146,572	7,757	0.007	0.474	0.030
Male	not-blocked	ABm non-blocked	7,126,054	148,350	0.021	0.762	0.280	6,379,722	60,105	0.009	0.895	0.405	544,629	4,998	0.009	0.076	0.034
Male	single oligo	AGT blocked	8,289,491	688,343	0.083	0.789	0.721	7,163,697	182,328	0.025	0.864	0.265	219,388	3,845	0.018	0.026	0.006
Male	not-blocked	AGT non-blocked	34,723,790	350,697	0.010	0.991	0.760	34,384,995	133,635	0.004	0.990	0.381	16,429	1,171	0.071	0.000	0.003
Male	multiple oligos	AG blocked replicate 1	31,007,664	515,303	0.017	0.748	0.315	27,316,990	132,428	0.005	0.881	0.257	2,366,143	1,920	0.001	0.076	0.004
Male	multiple oligos	AG blocked replicate 2	28,363,549	460,204	0.016	0.662	0.262	25,543,687	134,655	0.005	0.901	0.293	1,614,054	1,986	0.001	0.057	0.004
Male	not-blocked	AG non-blocked replicate 1	38,746,840	91,014	0.002	0.951	0.441	38,409,982	72,484	0.002	0.991	0.796	191,181	757	0.004	0.005	0.008
Male	not-blocked	AG non-blocked replicate 2	48,155,761	186,294	0.004	0.969	0.447	47,916,918	107,491	0.002	0.995	0.577	132,135	948	0.007	0.003	0.005

**Table 2:** Annotation analysis of rRNA component (18S, 5.8S, 2S and 28S) mapping reads, for the blocked and non-blocked sRNA-seq libraries. Shown for each sample are the number of redundant (R) and unique non-redundant (NR, unique) reads, the overall complexities (ratio of NR:R) and the proportion (Prop) of R and NR reads incident with each annotation. HT = head+thorax; AB = abdomen (subscripts f and m for female and male, respectively); AGT = male accessory glands + testes; AG = male accessory glands.

			18S rRNA matching reads					5.8S rRNA matching reads					2S rRNA matching reads					28S rRNA matching reads				
Sex	Treatment	Sample	R	NR	C	Prop R	Prop NR	R	NR	C	Prop R	Prop NR	R	NR	C	Prop R	Prop NR	R	NR	C	Prop R	Prop NR
Female	not-blocked	HTf non-blocked	75,339	13,401	0.178	0.007	0.107	40,868	868	0.021	0.004	0.007	4,429,692	101	0.000	0.397	0.001	181,519	25,319	0.139	0.016	0.201
Female	not-blocked	ABf non-blocked	119,038	12,420	0.104	0.006	0.081	52,590	910	0.017	0.003	0.006	16,310,008	101	0.000	0.843	0.001	430,673	23,571	0.055	0.022	0.154
Male	single oligo	ABm blocked	161,920	20,277	0.125	0.067	0.077	36,107	1,241	0.034	0.015	0.005	7,691	42	0.005	0.003	0.000	403,840	38,619	0.096	0.167	0.147
Male	not-blocked	ABm non-blocked	76,353	16,567	0.217	0.011	0.112	14,374	1,021	0.071	0.002	0.007	5,855,395	105	0.000	0.822	0.001	188,681	31,036	0.164	0.026	0.209
Male	single oligo	AGT blocked	2,065,077	23,744	0.011	0.249	0.034	571,755	1,365	0.002	0.069	0.002	1,991	44	0.022	0.000	0.000	3,978,074	45,811	0.012	0.480	0.067
Male	not-blocked	AGT non-blocked	824,905	24,676	0.030	0.024	0.070	369,597	1,323	0.004	0.011	0.004	29,922,115	87	0.000	0.862	0.000	1,423,871	47,224	0.033	0.041	0.135
Male	multiple oligos	AG blocked replicate 1	9,256,976	32,830	0.004	0.299	0.064	324,952	1,495	0.005	0.010	0.003	12,535	47	0.004	0.000	0.000	16,924,417	63,471	0.004	0.546	0.123
Male	multiple oligos	AG blocked replicate 2	8,341,130	33,250	0.004	0.294	0.072	406,771	1,620	0.004	0.014	0.004	224,866	110	0.000	0.008	0.000	15,840,278	65,265	0.004	0.558	0.142
Male	not-blocked	AG non-blocked replicate 1	810,082	22,369	0.028	0.021	0.246	324,140	1,189	0.004	0.008	0.013	33,885,124	104	0.000	0.875	0.001	1,792,733	43,244	0.024	0.046	0.475
Male	not-blocked	AG non-blocked replicate 2	806,644	30,826	0.038	0.017	0.165	329,035	1,639	0.005	0.007	0.009	43,219,134	102	0.000	0.897	0.001	1,785,630	59,908	0.034	0.037	0.322

### Use of a single blocking oligo reduced the proportion of reads mapping to 2S rRNA

**Fig 2:**
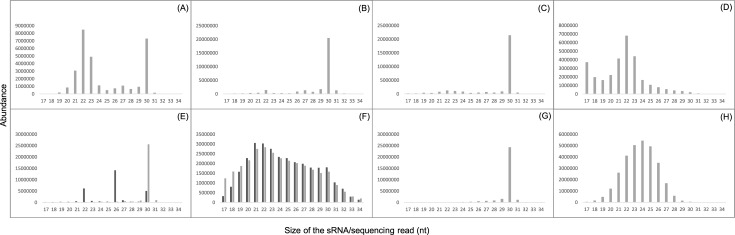
Size class distributions of redundant sRNA genome-matching reads for blocked and non-blocked samples. Samples shown are: (A) HT_f_ non-blocked; (B) AB_f_ non-blocked; (C) AB_m_ non-blocked; (D) AB_m_ single oligo blocked; (E) AG non-blocked (2 biological replicates shown in light and dark grey); (F) AG multiple oligo blocked (2 biological replicates shown in light and dark grey); (G) AGT non-blocked; (H) AGT single oligo blocked. Mapping was done for full length reads with 0 mis-matches and 0 gaps. For the HT_f_ sample (A) the bimodal distribution corresponded to the miRNA population (22nt peak) and rRNA fragments (30nt peak). For the AB_f_ sample a peak of 30mers was observed (comprising almost exclusively the 2S rRNA). The effectiveness of the single oligo blocking is shown by a comparison of panels C and D in the AB_m_ samples, with the blocked sample D comprising a 22mer peak of miRNA sequences, which was not previously evident in C. The effectiveness of the multiple oligo blocking is shown by a comparison of panels E versus F, with the blocked F revealing a unimodal, rich distribution with a peak at 22nt rather than the few dominant sequences shown in the unblocked E. For male AGT samples, the single oligo blocking approach (panel H) eliminated the 2S rRNA (30mer) that was dominating in panel G. However, this time the resulting distribution in the blocked sample (panel (H) was centred on a mode of 24nt, comprising additional rRNA fragments, with the 22mer miRNA population not forming second peak in this case.

The dominant representation of 2S rRNA-mapping reads in the AB_f_ body part, versus the HT_f_ sample (e.g. see Fig 2B versus Fig 2A, respectively), represented a significant problem for further characterisation and comparison of these sRNA populations. The sequencing output for the HT_f_ sample yielded an informative proportion of miRNA mapping reads. However, for the AB_f_ library, the reads aligning to the 2S rRNA swamped the sequencing space, resulting in a very low proportion of reads aligning to non-ribosomal sRNAs. It was not possible to exclude the 30nt 2S rRNA through size selection, due to the overwhelming abundance of this fraction. Therefore we explored the ‘blocking oligo’ method developed by [21], which is reported to deplete the 2S rRNA fraction without the need for additional magnetic beads, or RNase H steps.

We designed a 30 nt blocking oligo complementary to the 2S rRNA sequence, with 5' AC6 and 3' ddC modifications. By modifying both termini, we aimed to prevent both 3' and 5' adapter ligation to the oligos, and consequently any bound 2S fragments. The blocking oligo was introduced directly to the extracted RNA, without any prior size selection with PAGE gels (although the RNA extractions were enriched for fragments <200 nt). Having found a greater proportion of 2S rRNA in the AB_f_ compared to HT_f_ libraries, we subsequently used abdomen-derived tissues for testing the development of the blocking oligo protocol. RNA was extracted from a pool of 30 male abdomens (AB_m_) and 200 dissected pairs of accessory gland + testes (AGT). Two libraries were constructed from each extraction, using either the standard protocol without blocking oligos, or with the addition of the blocking oligo.

Consistent with the AB_f_ sample, AB_m_ and AGT libraries made using the standard protocol showed a similar, dominant 30nt insert band when visualised by gel electrophoresis (Fig 1B). The sequencing results from these samples also indicated a low complexity for the AB_m_ library of 0.057, and an even lower complexity of 0.013 for AGT samples. The annotation of reads revealed 89.5% and >99% of reads aligned to rRNA in AB_m_ and AGT samples, respectively. For the AGT samples, this resulted in an extremely low proportion of miRNA mapping reads (<0.001%). In agreement with the gel images, size class distributions of redundant reads confirmed the majority of reads were 30nt in length (Fig 2G) and >80% of reads aligned to the 30 nt 2S rRNA in each sample.

Strikingly, when the blocking oligo was used in AB_m_ library construction, the proportion of 2S rRNA aligning reads was reduced from 82% to 0.003% of all genome-matching reads. Consequently, the total proportion of rRNA mapping reads fell to 28.1%, while almost half of all genome matching reads aligned to miRNAs (Table 1, Table 2). For the blocked AGT library, reads mapping to 2S rRNA were similarly reduced to <0.001% of the total. However, despite this reduction, and in contrast to the blocked AB_m_ libraries, total rRNA reads remained relatively high at 86.4%, due to the presence of additional rRNA fragments. The proportion of miRNA incident reads in these samples was increased in comparison to the standard AGT library, but at just 2.6% this low proportion could still represent a challenge for robust comparisons of sRNA abundances.

### 2S rRNA blocking revealed additional, specific and abundant rRNA fragments requiring multiple rRNA blocking

**Fig 3:**
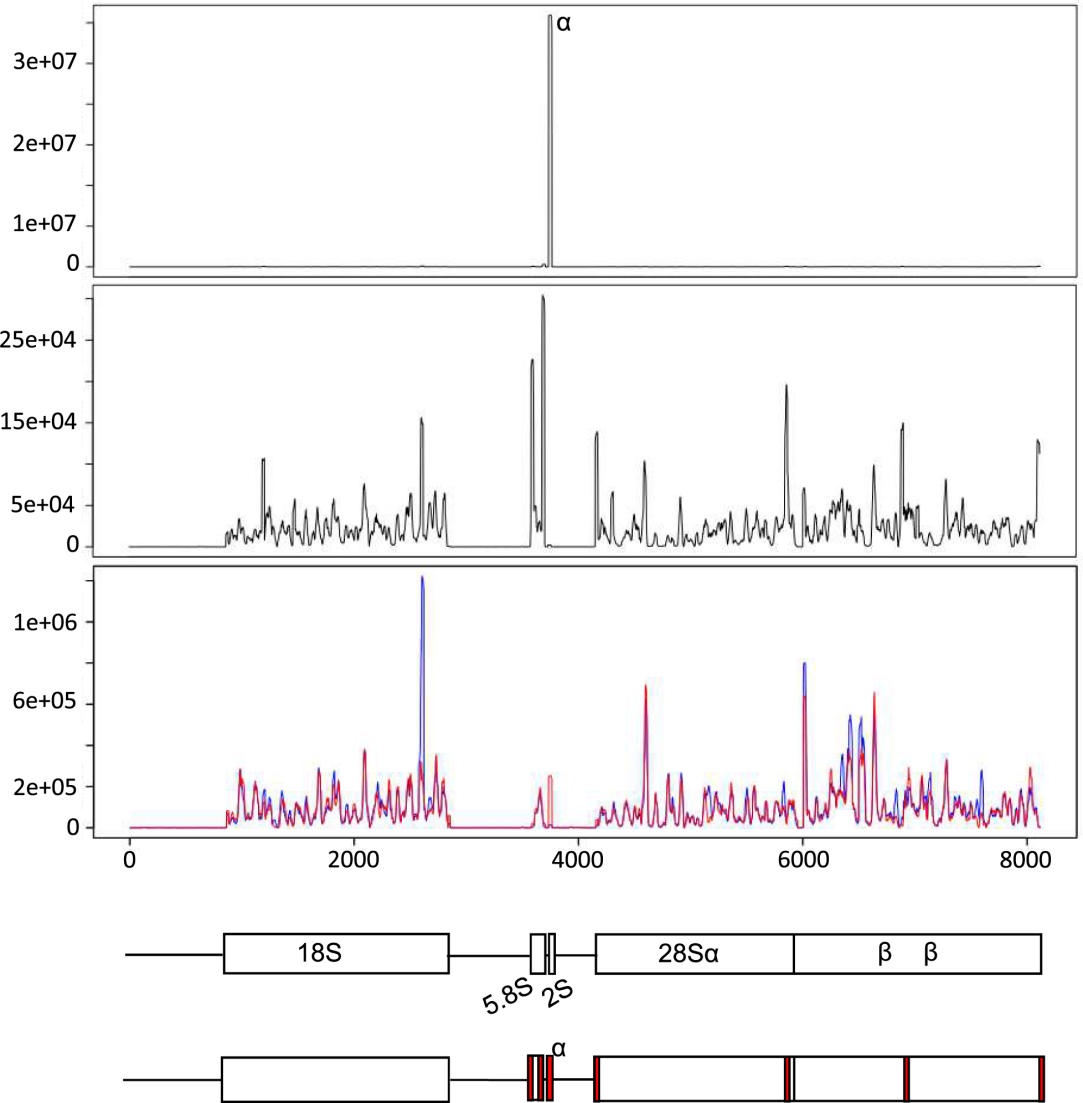
Distribution of rRNA incident reads. Pre-rRNA presence plots were obtained using perfect sRNA matches on the pre-rRNA transcript. Shown are the presence plots for (A) AGT not blocked; (B) AGT single oligo blocked; (C) AG multiple oligo blocked (2 biological replicates are presented in red and blue). The location of the blocking oligos is indicated by the numbered labels 1–7. For the presence plots, on the x-axis we represent the location across the transcript, on the y-axis, we represent (in linear scale) the sum of the un-normalized abundances of incident sRNAs with any given position. Shown at the bottom of the figure is the structure of the pre-rRNA transcript, indicating the location of the blocking oligos in red.

To investigate the identity of the other abundant rRNA fragments in the AGT sequencing libraries subjected to the single oligo blocking, we examined the identity of the reads aligning to *D*. *melanogaster* rRNA sequences (Fig 3A and 3B). The abundant peak corresponding to the 2S gene in the standard AGT library (Fig 3A) was almost completely absent in the blocked library (Fig 3B), demonstrating that the single oligo blocking was highly efficient. In the blocked libraries, reads were distributed along the 18S, 5.8S and 28S genes, with spikes in abundance at distinct locations. Interestingly, there was an enrichment for reads aligning to the 3’ and 5’ termini of both the 5.8S and 28S genes and this was notably absent for 18S. These results suggested that some of the abundant rRNA fragments in the blocked AGT sequencing were specific, rather than random degradation products, and could therefore be targeted for further depletion using blocking oligos.

To test whether we could reduce the level of rRNA fragments in reproductive tissue samples further, and hence increase the proportion of miRNA-aligning reads, we designed an ‘oligo cocktail’ to block the most abundant rRNA fragments present in the AGT samples (S2 Table). The cocktail consisted of 8 oligos, complementary to the 2S rRNA, the 6 most abundant fragments aligning to the pre-rRNA (Fig 3) and a single fragment of the 3’ region of the 5S rRNA. To test the oligo cocktail, two replicate pools of enriched RNA were extracted from male accessory glands (AG). These extractions were each split into two and libraries were constructed using either the standard library protocol (no blocking oligos), or with the addition of the oligo cocktail. The standard and blocked libraries were PCR amplified, and separated on 8% PAGE gels (Fig 1C). For the standard AG libraries, both replicates showed the 30 nt band of 2S rRNA as seen in the other tissue libraries made without a blocking oligo. However, in replicate 1, two shorter bands were also visible. Subsequent sequencing of these libraries revealed peaks in abundance of 22 and 26 nt fragments (Fig 2E), and the majority of those reads were annotated as 2S rRNA. It is not clear why, in this replicate, distinct 22 and 26 nt 2S rRNA fragments were present. However, it is important to note that these intense bands were absent in the oligo cocktail treatment, hence the gel electrophoresis indicated targeted fragments had indeed been successfully blocked. Libraries were extracted from the gel, normalised and pooled for sequencing.

The sequencing results revealed that for both replicates, the oligo cocktail was effective at blocking all rRNA target sequences, as shown by the absence of peaks in the targeted regions in Fig 3C. The proportion of 2S rRNA was reduced from >87% of the total redundant reads to <0.01% in the blocked samples. Overall, the proportion of redundant reads annotated as rRNA fell from >99% to ~90% when the oligo cocktail was used. The high proportion of rRNA remaining was mainly attributed to a large increase in reads aligning across the 18S, and untargeted regions of the 28S (~30% and >50% of the total, respectively) in the blocked libraries. The proportion of reads annotated as miRNAs increased 10-fold to 7.6% and 5.7%, in replicate 1 and 2, respectively. This was an improvement on the 2.6% of miRNAs resulting from the use the single blocking oligo and represented an increase in abundance of sRNAs to a level that could be used for quantitative analysis of sRNA populations.

### Using multiple blocking oligos did not alter the pattern of miRNA expression

It was important to evaluate whether the blocking oligos influenced the composition of the sRNA/miRNA population in the different tissues, to rule out any potentially confounding bias introduced by the blocking procedure. To do this, we compared the miRNA abundances between blocked and non-blocked libraries using Bland-Altman MA plots (S1 Fig) and correlation analyses (S2 Fig).

In the MA plots, each point corresponds to an expressed sRNA (the rRNA fragments were excluded). Reproducible, comparable samples are characterized by a typical ‘funnel-like’ pattern (e.g. Panel A in S1 Fig) resulting from high consistency (in identity, rank and abundance) between the abundant reads and lower consistency for less abundant reads. In contrast, less comparable samples typically show a dispersed MA plot (e.g. Panel B in S1 Fig), resulting from low numbers of ‘usable’ reads or from noise. The AB_m_ samples (Panel A in S1 Fig) showed good concordance between the unblocked and blocked samples. The AGT samples (Panel B in S1 Fig) showed lower concordance, due to the small number of miRNAs present in the non-blocked samples. For the AG samples, we first compared the biological replicates (Panel C in S1 Fig, non-blocked; Panel D in S1 Fig, blocked). We observed good reproducibility for the miRNAs in the blocked samples, with a ‘tight’ (funnel-shaped) MA plot at higher abundances and aligned around the 0 difference line. However, for the non-blocked libraries the MA plot showed a more dispersed distribution of abundances, with less tight funnelling and alignment around 0. This dispersed distribution was a direct consequence of the smaller proportion of reads assigned to miRNAs and overall lower abundance of these reads in the first non-blocked replicate. When comparing the blocked versus non-blocked AG libraries we observed that for the second replicate of the non-blocked libraries the sRNA populations were more consistent in comparison to the blocked libraries (Panel G in S1 Fig versus Panel H in S1 Fig), similar to the AB_m_ samples. However, in the first replicate of the unblocked versus the blocked libraries there was a more dispersed MA plot (due to the small number and reduced abundance of miRNAs).

We then conducted a quantitative analysis using correlation coefficients (Pearson (PCC), Spearman (SCC), Kendall (KCC)) to evaluate the similarity between the abundance and ranking levels of the sRNA reads within the blocked versus non-blocked libraries (S2 Fig). For the AB_m_ samples, the Pearson correlation (black solid line) was consistently above 0.9 (with SCC and KCC also high) indicating tight abundance- and rank-based correlations between these samples. For the AGT samples, the dispersion observed in the MA plot was evident in low correlation coefficients—as abundance increased, the correlation between the blocked and unblocked decreased towards 0.7 for PCC, 0.4 for SCC and 0.3 for KCC. The low values for the SCC and KCC indicated a high variability in the ranking of the miRNAs, which, linked with the higher PCC, suggested that most miRNAs were found within a narrow, low abundance range. Similarly, the correlation between the AG non-blocked samples was in the lower range, whereas the correlation between the blocked samples was consistently high. As observed in the MA plots the correlations of the blocked samples with the second replicate of the non-blocked samples was high. For the first replicate of the unblocked libraries there was a lower correlation for low abundance reads, although this increased as the abundance threshold increased. This suggested that most of the variability was observed in the low abundance range, potentially resulting from the presence or absence of reads at the noise:signal threshold). High consistency between the miRNA populations in the blocked versus non-blocked was observed for the highly abundant miRNAs.

The correlation analyses and MA plots showed that that the single oligo blocking was efficient for the AB samples, but not for individual tissues. For the AGT, the multiple blocking oligo treatment was successful and yielded highly reproducible sRNA libraries in which the identity and abundance ranking of the miRNA population remained unchanged. Non-blocked libraries tended to have more dispersed MA plots when only miRNAs are represented because the rRNA fragments occupy most of the sequencing space, leaving little opportunity to correctly reflect the miRNA abundances themselves. A computational exclusion of rRNA-annotated reads would not solve this problem given the limited sequencing space assigned to the miRNA class. However this can be fixed by blocking rRNAs experimentally, from libraries prior to the sequencing itself, as we did in this study.

## Discussion

In this study, we developed and applied an effective approach for the depletion of rRNA fragments from tissue-specific sRNA-seq libraries using a selective ‘blocking oligo’ method. We adapted the approach of [21] by: i) modifying the blocking oligos on both the 5’ and 3’ ends, to prevent ligation of each adapter; ii) adding the blocking oligos directly to the extracted RNA, prior to size selection, to further simplify the application of the approach to different protocols, and iii) developing and applying a novel extension to the protocol, based on a selective ‘oligo cocktail’, designed to target multiple abundant rRNA fragments.

The results showed that the use of blocking oligos was a highly effective and specific method of eliminating problematic rRNA sequences from library construction. Each blocking oligo typically reduced the 2S rRNA target sequences from >80% to < 0.01% of the total read number, crucially without altering the underlying miRNA profile. Importantly, we showed that the use of multiple blocking oligos can amplify miRNA abundances (e.g. Fig 2E versus 2F) facilitating informative comparisons of sRNA populations within tissues that were previously resistant to such analyses because of rRNA contamination.

Of the samples tested in this study, male reproductive tissue had a particularly high ratio of rRNA to miRNAs and hence represented a challenge for analyses of sRNA populations. The existence of variation in the ratio of rRNAs is consistent with previous research [22] and likely reflects differences in biological activity across tissues. For example, a high level of rRNA in male accessory glands may be required to produce and replenish the >130 proteins secreted into the ejaculate from these structures [23]. The rate of rRNA and ribosomal protein synthesis in accessory gland tissues increases following copulation [24]. Hence, mated males (as used in this study) may have a higher proportion of rRNA in reproductive tissues than virgin males. The roles of sRNAs in regulating the expression of genes in such tissues is of much interest and the preparation of RNA-seq libraries from specific tissues for these analyses is highly advantageous to avoid signal swamping by neighbouring tissues and to enable a high resolution description of differential expression [14]. However, as we observed here, tissue-specific RNA-seq can be compromised by the presence of a high proportion of rRNA matching reads. The methods we adopted and developed successfully addressed this problem and allow the analyses of sRNA populations in different types of tissues with varying biological roles.

We found that single oligo blocking of 2S rRNA from abdomen tissue samples increased the proportion of miRNA-mapping reads (e.g. Fig 2C versus 2D) and caused a dramatic reduction in the abundance of the targeted sequences. However, this procedure was not as effective in specific reproductive tissues (accessory gland and testes). For these, we found that the depletion of 2S rRNA enhanced the proportion of sequencing space allocated/assigned to other rRNA fragments. There was a distinct enrichment for these additional, specific rRNA sequences: notably, the 5’ and 3’ termini of the 5.8S and 28S species (but not the 18S), which is a feature conserved in other animals [25,26]. The rRF5 and rRF3 terminal fragments we identified in this analysis are thought to have biological functions in the control of cell proliferation and apoptosis and hence may represent more than degradation products of rRNA processing [25]. In this manner, RNA sequencing has facilitated the annotation of many rRNA-derived small RNAs (srRNAs) with novel biological functions [27–29]. Hence sRNA-seq can enable the study of srRNA expression in its own right as well as identify particularly abundant srRNAs as potential targets for blocking during sequencing library construction. The approach we followed here could also be applicable to rRNA blocking in different taxa characterised by different srRNA fragments that might otherwise reduce the proportion of miRNA-mapping reads and challenge quantitative sRNA analyses.

In conclusion, we gained a deeper understanding of the expression profiles of rRNAs, which enabled the design of multiple blocking oligos to selectively decrease the abundance of rRNA fragments and increase the amount of useful information from our sequencing experiments. This simple, cost effective technique can enhance the proportion of miRNA-mapping reads in tissues with high rRNA:sRNA ratios, while preserving the underlying miRNA population profile. Increasing the number and identity of sRNAs present in the datasets for specific tissues may increase the accuracy of differential expression analyses [30] and approaches for the identification and characterization siRNA loci in general [31].

## Materials and methods

### RNA extraction

*D*. *melanogaster* wild-type flies (Dahomey) were reared under standard conditions in which larvae were placed 100 per vial on SYA medium (15g Agar, 50g sugar, 100g brewer’s yeast, 30ml Nipagin (10% w/v solution), 3ml propionic acid per litre of water). Females were collected and kept in groups of 10 virgins per vial until they were mated to wild type males 5–7 days later and then flash frozen in liquid N_2_. Male flies were collected under CO_2_ anaesthesia, flash frozen and dissected on dry ice or in Phosphate Buffer Solution. Abdominal tissue from 30 males, and reproductive tissue from 200 males were pooled and stored at -80°C until use. For sRNA-enriched extractions, tissues were homogenised by grinding under liquid nitrogen. RNA containing fragments <200nt were extracted using the miRvana kit (Ambion, AM1561) according to the kit protocol and eluted in RNA Storage Solution (Ambion, AM7000). The quantity and quality of RNA extractions was measured using a NanoDrop 8000 spectrophotometer.

### Sequencing library construction

sRNA libraries were constructed using HD adapters [7] as in [8] with minor adjustments. Libraries were made following the standard protocol or with the addition of one blocking oligo complementary to 2S rRNA, or multiple blocking oligos complementary to the 5’ and 3’ ends of abundant processed rRNAs (full protocol in S1 Methods; oligo sequences in S3 Table).

### Illumina sequencing

All sRNA libraries were sequenced on an Illumina HiSeq 2500 platform, using a single-end, 50 nt read metric (sequencing providers BaseClear B.V. and The Earlham Institute). In total, we sequenced 1 HT_f_ and 1 AB_f_ samples, 2 AB_m_ samples, 2 AGT samples (blocked with a single 2S oligo vs. not blocked) and 4 AG samples (not blocked, or blocked with multiple oligos x 2 replicates each).

### Bioinformatics analysis

The sequencing fastq files were converted to fasta format and reads without Ns were retained for further analysis. The evaluation of quality scores was conducted as in the FastQC suite. The 3' adapter and HD signatures (4 assigned nt at the 3’ and 5’ end of the insert [7]) were trimmed using perfect string matching on the first 7 nucleotides of the adapter (TGGAATT). Next, the files were converted from redundant to non-redundant format and the results were summarised into redundant and non-redundant size class distributions [30].

In non-redundant format, the reads were mapped to the reference genome (*D*. *melanogaster* v 6.11) and associated annotations, allowing 0, 1 or 2 mis-matches and 0 gaps using PatMaN [32,33]. The reads were also mapped to mature miRNAs and miRNA hairpins, retrieved from miRbase, release 21 [19]. The sRNA analysis was conducted using the UEA sRNA Workbench, custom-made Perl and R scripts. The presence plots were created in R, v 3.4.0.

### Data access

The data presented in this study are publicly available on Gene Expression Omnibus [34] under accession numbers GSE86313 (male AB samples), GSE98833 (male AGT and AG samples) and GSE99673 (female HT and AB samples).

## Supporting information

S1 Methods Small RNA library protocol with blocking oligos for *Drosophila melanogaster*. (PDF)

S1 Table Overview table showing the sequencing characteristics of the blocked and non-blocked sRNA-seq samples. (XLSX)

S2 Table Non-normalized expression levels of miRNAs present in the blocked and non-blocked libraries. (XLSX)

S3 Table Sequences of the blocking oligos. (XLSX)

S1 Fig MA plots to compare genome-matching sRNA populations in blocked versus non-blocked samples. (PDF)

S2 Fig Correlation analyses (Pearson (PCC), Spearman (SCC) and Kendall (KCC) correlation coefficients) for the miRNA expression levels in blocked versus non-blocked samples. (PDF)
