# Supplementary material for: Small RNA populations revealed by blocking rRNA fragments in Drosophila melanogaster reproductive tissues
Source: PLoS One. 2018 Feb 23;13(2):e0191966. doi: 10.1371/journal.pone.0191966 (PMC5825024; doi:10.1371/journal.pone.0191966)
Supplement: S1 Methods — (PDF) [file pone.0191966.s001.pdf]

S1 Methods - Small RNA library protocol with blocking oligos for *Drosophila melanogaster*

(i) Using the blocking oligos, and ligating 3' adapter

- Use at least 1 µg of RNA sample, in no more than 10 µl.
- Use 10 pmol of blocker oligo (so it is the equivalent amount to 3' adapter), and add this before 3' adapter ligation.

Note: if 3' adapter stock is not already adenylated, refer to section xii

**Add blocker. Mix in 0.2 ml PCR tube:**	
RNA sample	10 µl
Blocker oligo (at 10 µM concentration)	1 µl
NEB T4 RNA ligase buffer	2 µl
Riboguard RNase inhibitor	0.75 µl
PEG8000 (warm to 37°C first to get rid of cloudiness, then keep at room temperature)	4 µl
Incubate in PCR machine: 70°C for 2 mins, 60°C for 5 mins	
**Ligate 3' adapter. To the above mix, add:**	
pre-adenylated 3' adapter (<u>see section xii details</u>)	10 pmol (volume depends on concentration)
NEB truncated T4 RNA ligase	1 µl
Incubate in the PCR machine: 26°C for 2 hours	

(ii) Clean up using Zymo kit

Use RNA cleaning and concentrating kit (Zymo, R1018 – Cambridge Bioscience) to clean up reaction.

**In 0.5 ml tube:**
Make volume up to 50 µl total with H_2O
Add 100 µl RNA binding buffer
Add 150 µl 100% ethanol
Transfer to spin column and spin at 12,000 rpm for 1 minute
Discard flow-through and add 400 µl RNA prep buffer. Spin at 12,000 rpm for 1 minute

Discard flow-through and add 800 µl RNA wash buffer and spin at 12,000 rpm for 30 seconds
Repeat this wash step with 400 µl wash buffer
Discard flow-through and spin at 12,000 rpm for 2 minutes
Transfer column to clean tube and elute with 13 µl H_2O by spinning at 12,000 rpm for 1 minute.

(iii) Removing the 3' adapter

Add the following to the **12.1 µl eluted RNA** in a 0.2 ml PCR tube:

**Mix in a 0.2 ml tube:**	
Eluted RNA	12.1 µl
10x deadenylase buffer (available from Cambio, DA11101K)	1.6 µl
100 mM DTT	0.8 µl
Riboguard RNase inhibitor (available from Cambio, RG90925)	0.5 µl
Scriptminer finishing enzyme	1 µl
**Incubate at 30°C for 30 minutes**	
**Add 4 µl Scriptminer stop solution**	

Now mix the above reaction with the following:

**3' adapter degradation:**	
Scriptminer degradase buffer (OR 500 mM Tris-HCl pH9.0)	2 µl
Scriptminer $MgCl_2$	7 µl
Scriptminer degradase enzyme	1 µl
**Incubate at 37°C for 30 minutes**	

The entire product of this reaction is to be used in the next 5' adapter ligation step.

(iv) 5’ adapter ligation

Use a total of 20 pmol of 5’ adapter per reaction.

**Denature the 5’ adapter by heating at 70°C for 2 minutes, then place on ice.**	
Entire RNA sample following the previous degradase reaction	30 µl
Scriptminer 5’-RNA ligation buffer	1 µl
10 mM ATP	1 µl
Denatured 5’ adapter (10 µM)	2 µl
Scriptminer 5’ RNA ligase	1 µl
50% PEG	7 µl
**Incubate reaction at 26°C for 2 hours**	
**Add 8 µl H_2O to make up the total volume to 50 µl**	

(v) Clean up using Zymo kit

Transfer the 50 µl of ligated sample to a 0.5 ml tube
Add 100 µl RNA binding buffer
Add 150 µl 100% ethanol
Transfer to Zymo spin column and spin at 12,000 rpm for 1 min
Discard flowthrough
Add 400 µl RNA prep buffer and spin at 12,000 rpm for 1 min
Discard flowthrough
Add 800 µl RNA wash buffer and spin at 12,000 rpm for 30 secs
Repeat wash step with 400 µl RNA wash buffer
Discard flowthrough and spin at 12,000 rpm for 2 minutes
Transfer column to an RNase free tube
**Elute the samples TWICE using 15 µl H_2O each time (to end up with ~30 µl sample)**

(vi) cDNA synthesis

The RNA is now tagged with 3' and 5' adapters. To convert to cDNA mix together the following:

**di-tagged RNA sample**	**30 µl**
MMLV reverse transcription buffer	4 µl
dNTP PreMix	2 µl
DTT	2 µl
RTP primer	1 µl
Scriptminer MMLV reverse transcriptase	1 µl
**Incubate at 37°C for 20 minutes**	
**Terminate by incubating at 85°C for 15 minutes, then keep on ice.**	

(vii) PCR Amplification, round 1

Use 4 µl of the cDNA to run a 20 µl PCR reaction.

Note: for each different sample, *use a unique index primer*

For each sample, run 3 different PCR cycle numbers in order to optimise the reaction. As a guide, use 5, 7 and 9 cycles for the first attempt (if there is no contaminating 30mer band, the cycle number can be increased).

	**1 reaction**
H_2O	9.3 µl
10 mM dNTPs	0.5 µl
5x high fidelity Phusion buffer	4 µl
Illumina RP1 primer (10 µM)	1 µl
Illumina index primer (10 µM)	1 µl
cDNA	4 µl
Phusion high fidelity DNA polymerase (*NEB biolabs* #M0530S)	0.2 µl

(viii) Run PCR reaction on 8% PAGE gel

**8% PAGE gel**			
	**2 gels**	**4 gels**	**6 gels**
H_2O	10 ml	20 ml	30 ml
40% (19:1) acrylamide/bis solution	3 ml	6 ml	9 ml
5x TBE	1.5 ml	3 ml	4.5 ml
10% ammonium persulphate	150 µl	300 µl	450 µl
TEMED	7.5 µl	15 µl	22.5 µl

- Use one gel per sample.
- Mix 20 µl PCR product with 5 µl 5x Novex loading dye.

- Load a total of 20 µl in each well, and then mix what is left over from each tube and load that in a fourth lane.
- Load 10 µl of 20 bp ladder (Jena Bioscience) either side of the sample lanes.
- Run the gel in 0.5x TBE buffer for 2-2.5 hours at 120V
- Stain the gel with SYBR gold (5 µl in ~50 ml of 0.5x TBE) and scan.
- Print the gel images off in real-size.

(ix) Gel Extraction

- Prepare 0.5 ml tubes by punching 4 holes in the bottom of each with a 21 gauge needle. Put each 0.5 ml tube inside a 2 ml "collection" tube. Use one tube for each gel (or sample).
- Lay the gels over the real-size print-outs and use a razor blade to cut out the area containing the band of interest (re-scan the gels afterwards to check the correct area has been excised).
- Put the slice from each gel into a prepared 0.5 ml tube. Spin the tubes at max speed for 3 minutes to shred the gel slice.
- Discard the 0.5 ml tube. Add 400 µl NEB2 buffer to the broken gel and incubate overnight, shaking at 4ºC.
- Following the overnight incubation, transfer the gel mixture to a Spin-X column (0.45 µm, *ThermoFisher*), and spin at 2800 RPM for 3 mins, to remove gel debris.
- To 400 µl eluate, add 2 µl glycogen, 40 µl of 3M sodium acetate, and 1200 µl 100% ethanol
- Incubate at -80ºC for 20-30 minutes
- Spin at 4ºC, 20000 RPM for 20 minutes
- Remove supernatant, and wash the pellet in 500 µl 70% ethanol
- Spin at room temp, 13000 RPM for 2 mins
- Remove supernatant and re-suspend pellet in 13 µl H_2O

(x) PCR amplification, round 2

Take **1 µl** of the gel extraction to run another PCR, again with the 3 different cycle numbers:

	**1 reaction**
H_2O	12.3 µl
10 mM dNTPs	0.5 µl
5x high fidelity Phusion buffer	4 µl
Illumina RP1 primer (10 µM)	1 µl
Illumina index primer (10 µM)	1 µl
cDNA	1 µl
Phusion DNA polymerase	0.2 µl

- Following this 2nd round of PCR, decide which cycle number gives you the best band. This cycle number needs to be used in the final amplification.
- Repeat the gel extraction as detailed above.

(xi) Final PCR

Set up the final PCRs, using the chosen cycle number for each sample, and set up 7 identical reactions per sample:

	**1 reaction**
H_2O	12.3 µl
10 mM dNTPs	0.5 µl
5x high fidelity Phusion buffer	4 µl
Illumina RP1 primer (10 µM)	1 µl
Illumina index primer (10 µM)	1 µl
cDNA	1 µl
Phusion DNA polymerase	0.2 µl

- Run all 7 of the reactions from each sample on one gel, loading the spare 8th lane with the leftovers.

- Once the gel has run, gel extract across all 8 lanes using the protocol above, and re-suspend the pellet in final volume of ~10-12 µl

(xii) Adenylation of the 3' HD adapter (if necessary)

Note: Also check that the 3' HD adapter stock is already phosphorylated.

Use 200 pmol of 3' adapter per 40 µl reaction (this should give enough 3' adapter to make about 20 libraries)

**Adenylate 3' adapter. Mix in a tube:**	
3' adapter	2 µl
10x 5' DNA adenylation reaction buffer (NEB #B2610S)*	4 µl
1 mM ATP (NEB #N0757A)*	4 µl
Mth RNA ligase (NEB #M2611A)*	4 µl
Nuclease-free H_2O	26 µl
Incubate for 1 hour at 65°C, then 5 mins at 85°C	
**Phenol chloroform extract the adapter:**	
Adenylated adapter	40 µl
H_2O	60 µl
Phenol chloroform	100 µl
Vortex and spin at 13000 rpm for 15 minutes	
Transfer aqueous layer to new tube	
**Ethanol precipitation:**	
Aqueous phase	Approx. 75 µl
H_2O	25 µl
Glycogen	2 µl
3M sodium acetate	10 µl
100% ethanol	250 µl
Incubate overnight at -20°C	
**Obtain pellet and wash:**	
Spin reaction at 13000 rpm for 20 minutes @ 4°C	
Remove supernatant	

Wash pellet with 500 µl 80% ethanol
Spin at 13000 rpm for 5 minutes
Remove ALL supernatant (allow to air for a bit if necessary, but be careful to not let pellet completely dry)
Resuspend pellet in 12 µl of Ambion RNA storage solution.

*ORDERED AS A KIT FROM NEW ENGLAND BIOLABS CAT# E2610S

Running 3' adapter on PAGE gel

**16% PAGE urea gel**	**For 1 gel:**	**For 2 gels:**
	**10 ml**	**15 ml**
Urea*	4.2 g	6.3 g
H_2O*	2.5 ml	3.5 ml
40% (19:1) acrylamide/bis solution	4 ml	6 ml
5x TBE	1 ml	1.5 ml
10% ammonium persulfate (APS)	100 µl	150 µl
TEMED	5 µl	7.5 µl

*Dissolve the urea in the H_2O first by heating in a falcon tube in the microwave for 10 second bursts at a time, then add remaining ingredients to this solution.

- To check for successful 3' adenylation, run adenylated 3' adapter on a PAGE gel (16% urea) against non-adenylated 3' adapter (the stock) and a Scriptminer control.
- Use ~1 pmol of adenylated and non-adenylated adapter. Also mix 1 pmol each of adenylated and non-adenylated together and run this between the other two samples.
- Run 0.5 µl of a 1:10 dilution of Scriptminer 3' adapter as a control.
- Load a total volume of 10 µl. Use 2x denaturing (formaldehyde) loading dye.
- Run at ~120V in 0.5x TBE for 2.5-3 hours.
- Stain the gel with SYBR gold and view using a scanner
- The adenylated adapter should be slightly larger than the non-adenylated. You should see two distinct bands in the mixed sample. The Scriptminer band should be smaller than the others.

- Note: You might see a smaller faint band in the ‘adenylated’ adapter sample, indicating some non-adenylated adapter remaining. This doesn’t matter, the adapter is still OK to use as long as most of it is adenylated.
