# Supplementary material for: Small RNA populations revealed by blocking rRNA fragments in Drosophila melanogaster reproductive tissues
Source: PLoS One. 2018 Feb 23;13(2):e0191966. doi: 10.1371/journal.pone.0191966 (PMC5825024; doi:10.1371/journal.pone.0191966)
Supplement: S1 Fig — (PDF) [file pone.0191966.s005.pdf]

S1 Fig

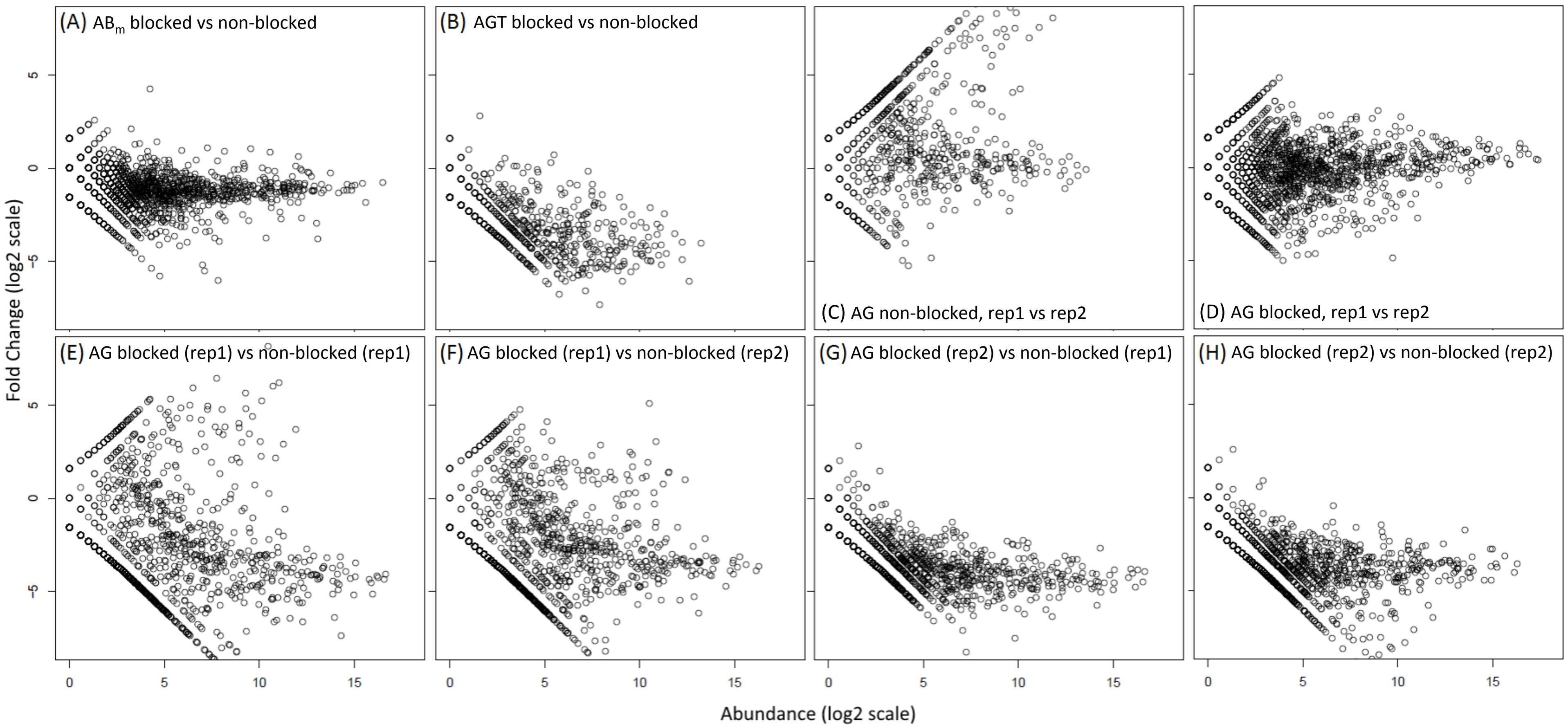
(A) AB_m blocked vs non-blocked
(B) AGT blocked vs non-blocked
(C) AG non-blocked, rep1 vs rep2
(D) AG blocked, rep1 vs rep2
(E) AG blocked (rep1) vs non-blocked (rep1)
(F) AG blocked (rep1) vs non-blocked (rep2)
(G) AG blocked (rep2) vs non-blocked (rep1)
(H) AG blocked (rep2) vs non-blocked (rep2)
Fold Change (log2 scale)
Abundance (log2 scale)
5
0
-5
0
5
10
15

S1 Fig. MA plots to show comparisons between sRNA populations in blocked versus non-blocked samples. The data shown are the non-normalized abundances of *D. melanogaster* sRNAs (reads matching to the genome, full length, with no mis-matches or gaps). For each point (sRNA) the average abundance, indicated on the x-axis (log_2 scale), is plotted against the differential expression between the two samples being compared on the y-axis (fold change difference, in log_2 scale). The plots show comparisons between pairs of blocked / non-blocked libraries as well as between replicates of the same treatment, as follows: (A) AB_m, blocked vs non-blocked, (B) AGT blocked vs non-blocked, (C) AG non-blocked, replicate 1 vs replicate 2, (D) AG blocked, replicate 1 vs replicate 2, (E) AG blocked (replicate 1) vs non-blocked (replicate1), (F) AG blocked (replicate 1) vs non-blocked (replicate 2), (G) AG blocked (replicate 2) vs non-blocked (replicate 1), (H) AG blocked (replicate 2) vs non-blocked (replicate 2). Panels A, D, G, H showed a ‘funnel’ shape of decreasing DE from low to high abundance and hence the replicates or samples compared in these plots were very similar to one another. Panels B, C, E, F show distributions that were more widely dispersed, less funnelled and hence indicative of samples or replicates that are less similar. These patterns suggest that the single oligo blocking performed well on AB_m samples (A), but less well on AGT samples (B), because the distribution of sRNA expression was not changed by the blocking procedure in the AB (A), whereas it was for the AGT samples (B). The multiple oligo cocktail produced two replicates that were very similar (D). Comparisons between replicate multiple oligo blocked and non-blocked samples (E-H), showed that, in general the distribution of sRNA abundances was not altered by the multiple oligo blocking (G, H). However, the small number of usable sRNAs (<16k) in replicate 1 of the AG non-blocked sample resulted in more dispersed MA plots in comparisons with the non-blocked samples (E, F). The plots tended to be centred below 0 fold change differential expression in all comparisons in which blocked versus non-blocked samples were compared (A, B, E-H). This indicated that the non-blocked samples in each case had a lower level of sRNA expression overall (i.e. a smaller proportion of the sequencing space was allocated to sRNAs). This was the problem we successfully addressed here by increasing the representation of sRNA reads within the blocked samples.
