# Supplementary material for: Small RNA populations revealed by blocking rRNA fragments in Drosophila melanogaster reproductive tissues
Source: PLoS One. 2018 Feb 23;13(2):e0191966. doi: 10.1371/journal.pone.0191966 (PMC5825024; doi:10.1371/journal.pone.0191966)
Supplement: S2 Fig — (PDF) [file pone.0191966.s006.pdf]

S2 Fig

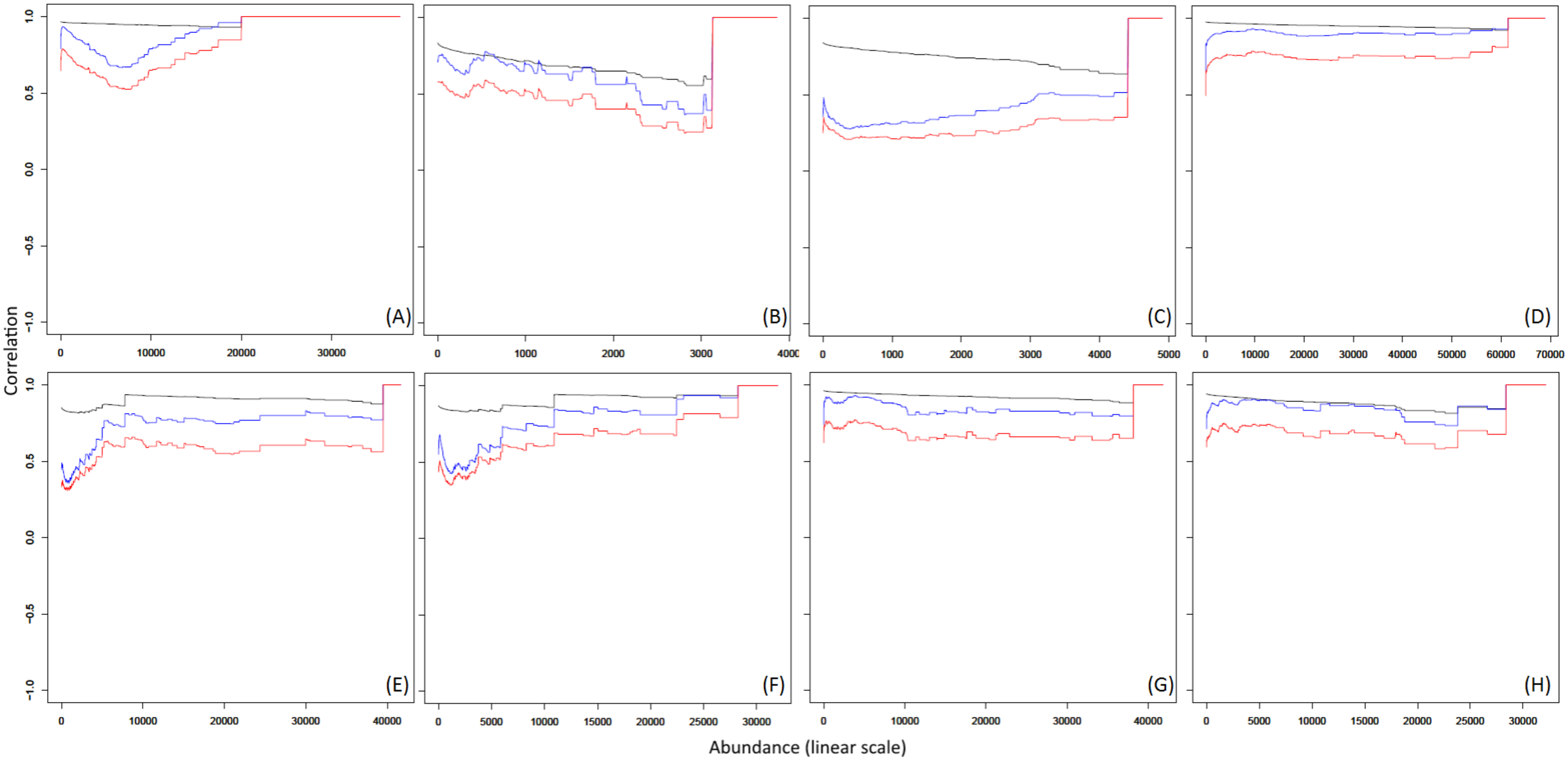

Correlation
Abundance (linear scale)
(A)
(B)
(C)
(D)
(E)
(F)
(G)
(H)
1.0
0.5
0.0
−0.5
−1.0
0
10000
20000
30000
40000
1000
2000
3000
4000
5000
50000
60000
70000
15000
25000

S2 Fig. Correlations (Pearson (PCC), Spearman (SCC) and Kendall (KCC) correlation coefficients) between the miRNA expression levels in blocked versus non-blocked samples. The input comprised of the non-normalized abundances of *D. melanogaster* miRNAs (mature sequences and their variants). The lines show the correlation coefficients (y-axis) between all miRNAs with abundances above a threshold indicated on the x-axis. The PCC is shown in black, the SCC in blue and the KCC in red. Parametric (Pearson) and non-parametric (Spearman and Kendall) correlation coefficients were used to determine whether the compared samples were similar both in distribution of abundances and ranking of the reads. The correlations coefficients were calculated on a minimum of 15 entries. The pairs of samples which were compared were: AB_m, blocked vs non-blocked (panel A), AGT blocked vs non-blocked (panel B), AG non-blocked, replicate 1 vs replicate 2 (panel C), AG blocked, replicate 1 vs replicate 2 (panel D), AG blocked (R1) vs non-blocked (R1) (panel E), AG blocked (R1) vs non-blocked (R2) (panel F), AG blocked (R2) vs non-blocked (R1) (panel G), AG blocked (R2) vs non-blocked (R2) (panel H). The single oligo blocking performed well on AB samples (panel A), but less well on AGT (panel B). The multiple oligo cocktail produced highly reproducible replicates (panel D) and preserved (and enhanced) the miRNA abundance distribution (panels E, H).
